# Resolving complex cartilage structures in developmental biology via deep learning-based automatic segmentation of X-ray computed microtomography images

**DOI:** 10.1038/s41598-022-12329-8

**Published:** 2022-05-24

**Authors:** Jan Matula, Veronika Polakova, Jakub Salplachta, Marketa Tesarova, Tomas Zikmund, Marketa Kaucka, Igor Adameyko, Jozef Kaiser

**Affiliations:** 1 Central European Institute of Technology, Brno University of Technology, Purkynova 123, Brno, 61200 Czech Republic; 2 Max Planck Institute for Evolutionary Biology, August-Thienemann-Str.2, 24306 Ploen, Germany; 3 Medical University of Vienna, Spitalgasse 23, 1090 Vienna, Austria; 4 Department of Physiology and Pharmacology, Karolinska Institutet, 17165 Stockholm, Sweden

**Keywords:** Cartilage development, X-ray tomography, Image processing

## Abstract

The complex shape of embryonic cartilage represents a true challenge for phenotyping and basic understanding of skeletal development. X-ray computed microtomography (μCT) enables inspecting relevant tissues in all three dimensions; however, most 3D models are still created by manual segmentation, which is a time-consuming and tedious task. In this work, we utilised a convolutional neural network (CNN) to automatically segment the most complex cartilaginous system represented by the developing nasal capsule. The main challenges of this task stem from the large size of the image data (over a thousand pixels in each dimension) and a relatively small training database, including genetically modified mouse embryos, where the phenotype of the analysed structures differs from the norm. We propose a CNN-based segmentation model optimised for the large image size that we trained using a unique manually annotated database. The segmentation model was able to segment the cartilaginous nasal capsule with a median accuracy of 84.44% (Dice coefficient). The time necessary for segmentation of new samples shortened from approximately 8 h needed for manual segmentation to mere 130 s per sample. This will greatly accelerate the throughput of μCT analysis of cartilaginous skeletal elements in animal models of developmental diseases.

## Introduction

To understand the complexity of embryonic development, it was essential to assess the shape and structure of tissues and organs in three-dimensional space. It also enabled us to dissect the sequential steps of their formation. Pioneering work introduced tissue contrasting techniques that enabled the detection of previously hidden structures such as embryonic cartilage or even their predecessor, mesenchymal condensations, using X-ray computed tomography^1–3^. The newly generated knowledge revolutionised the field of developmental biology and enabled, among others, the detection of the onset of congenital disorders and uncovering the origin and sequential steps of complex structure formation^4, 5^. During embryogenesis, the formation of the skull is preceded by the formation of chondrocranium. This cartilaginous 3D blueprint of future skeletal elements in the head is formed quite early in embryonic development and establishes the original layout of the future facial shape^6^. The shape of the head, specifically the face, is important for many aspects of everyday life—eating, breathing, vision, communication and mutual recognition in humans. Any morphological change in chondrocranium will be maintained even after replacement by bone. Therefore, when we aim to investigate the formation of the face, it is necessary to look at the embryonic stages and the 3D shape of the cartilage. Approximately 30% of congenital syndromes are represented by craniofacial malformations^7^. Investigations of the underlying causes were performed mainly using mouse genetic models that in part uncovered the basis of selected malformations. Nevertheless, numerous genetic perturbations were embryonically lethal and did not allow researchers to analyse and understand their role in the formation and shaping of embryonic structures.^8, 9^.

Historically, the investigation of head skeletal system formation relied on basic methodology such as histological staining of sections and a subsequent assembly of the 2D images into a stack^10^. Needless to say, this approach was prone to artifacts and time- and effort-demanding, not allowing us to unwind the 4D dynamics of face formation to the full extent or at high resolution. With the technological and contrasting advances in recent years, it has become possible to visualise nearly any structure in the developing embryo using 3D imaging techniques^2, 3^ and obtain more profound insights into the mechanisms of skeletal development, shaping and origin of craniofacial malformations. X-ray computed microtomography (μCT) is an imaging technique capable of capturing complex geometries in 3D with a high spatial resolution in the range of micrometres. This methodology became an ultimate booster in developmental biology, where the high spatial resolution allowed researchers to accurately assess the morphological properties of both hard and soft tissues of biological samples^11^. While advanced imaging protocols currently allow the detection of even delicate structures, such as embryonic cartilage shaping the face, the subsequent image processing preceding any further analysis remains enormously time-consuming and represents the major drawback of this methodology.

An essential step before any further analysis of μCT images is the segmentation of the structure of interest. Image segmentation is the task of assigning a class label to each pixel or, in the case of volumetric image data, the voxel of an image^12^. Many image segmentation algorithms have been developed and are actively utilised to segment mineralized matrices from μCT data. However, the low contrast of soft tissues (cartilage, peripheral nerves and others) represents a significant challenge for their application. High X-ray attenuation coefficients of hard tissues, such as bones and teeth, allow their segmentation with relative ease by applying simple segmentation algorithms, e.g., basic thresholding. Such image processing is unfeasible in the case of soft tissues^13^. The low X-ray attenuation provided by the various soft tissues present in biological samples renders them nearly transparent for X-rays with energies used in traditional laboratory μCT systems. Tissue contrasting with substances containing elements with high atomic numbers (iodine^14^, osmium^15^, tungsten^16^) is frequently used to enhance the visibility of soft tissues. The contrast between various soft tissues (for instance, peripheral nerves, cartilage, muscles or parts of the brain) results from the differential uptake of the contrast solution^17^. However, the generated contrast is insufficient for utilising traditional fully automatic segmentation algorithms. In many cases, the desired structures must be segmented manually due to the complex shapes and uncertain borders between different tissues. This manual segmentation is a taxing and time-consuming task, especially in the case of volumetric image data containing thousands of tomographic cross-sections. One such difficult-to-segment structure is the cartilaginous nasal capsule of a developing mouse embryo. 3D models created by manual segmentation were crucial in the work of Kaucka and colleagues.^2, 3^. Manual segmentation was a significant bottleneck in data processing in these publications, as the manual segmentation of cartilaginous nasal capsule in one μCT scan of a mouse embryo required at least 8 h of an expert’s time. Therefore, a fully automatic solution that could decrease the time requirement and manual work of the expert is highly sought after.

Deep learning and, specifically, convolutional neural networks (CNNs) consistently achieve state-of-the-art results in image segmentation tasks^18^. Therefore, they seem to be a logical candidate for automatic segmentation of the nasal capsule cartilage; however, there are several challenges. The µCT measurement provides extremely large image data (thousands of pixels in each plane). Such a high resolution cannot be compromised, as it is crucial in studies where minor morphological differences among several samples are sought and compared^2, 3^. Furthermore, the segmented cartilage is structurally inhomogeneous, and its shape differs considerably depending on its location within the embryonic head. Additionally, subtle intraspecies differences in cartilage geometry, structure and thickness are observed among individuals. The size of the training database also plays an important role in creating a robust CNN-based segmentation model.

U-Net is a well-established convolutional neural network architecture for the segmentation of biomedical images^12^. Its ability to learn from size-limited datasets stems from its fully convolutional nature with so-called skip connections and the lack of any fully connected layers. The success of the U-net architecture greately increased the popularity of so-called encoder-decoder architectures with skip connections in segmentation of biomedical images, where the encoder is responsible for feature extraction and the decoder for the localisation and segmentation of the desired structures. U-net’s power in segmentation of datasets with a limited training database stems from its fully-convolutional nature. In the work of Rytky and colleagues^19^ the authors propose a method for segmentation of calcified articular cartilage in µCT images of rabbit knees, where they utilise a feature pyramid network decoder with a ResNet-18^20^ encoder trained in the ImageNet dataset^21^. As articular cartilage is a relatively spatially homogeneous structure, the authors in^19^ can apply patch-based training with a relatively low input size. Similarly, the authors in the work of Léger and colleagues^22^ employ a 3-D U-Net CNN to segment mineralised cartilage in µCT images of the Achilles tendon-to-bone interface and can employ patch-based training due to the homogeneity of the segmented structure. To our knowledge, there is only one work dealing with a similar high-resolution segmentation of chondrocranium in developing mouse embryos imaged via µCT, published by Zheng and colleagues^23^. A manually annotated database is not available to the authors, and they approach the segmentation task using sparse annotation with uncertainty-guided self-training. The authors segment cartilage in the whole chondrocranium. They evaluated the performance of their method on selected, manually annotated subregions of the whole 3-D volume, as manual annotations of the whole chondrocranium were not available. This manual selection of the evaluation region may skew the final evaluation accuracy.

Here, we provide a methodology for fully automatic segmentation of highly complex cartilaginous nasal capsules in µCT images of mouse embryos. We utilised a CNN trained in a supervised training mode on a unique database of 14 manually annotated µCT scans of mouse embryo heads on their 17th day of embryonic development. We employed different modifications to a basic encoder-decoder CNN architecture to improve the segmentation performance of the model. We experimentally validated the proposed methodology for the particular image segmentation task. This µCT image segmentation model can be further used to segment newly scanned mouse embryos, thus greatly reducing the time required for processing new samples. The segmentation model is ready to be trained to include additional embryonic developmental stages or used as a basis for transfer learning for other high-resolution µCT segmentation tasks. We also show that the data provided by the proposed automatic segmentation methodology can be further quantitatively analysed in the same manner as manually segmented data.

## Methods

### Samples

The database for training and testing the proposed segmentation method consists of 14 micro-CT scans of mouse embryonic heads at E17.5 (developmental stage). The heads were contrasted using the PTA-staining procedure before scanning, which enabled the detection of tissues with low density (e.g., cartilage and muscle)^24^. The staining protocol was previously described in^2, 3, 25^. A subset of the dataset was published and is available for inspection in^26^. All samples utilised in this work are summarised in Suppl. Table S1. All animal work was approved by the Local Ethical Committee on Animal Experiments (Norra Djurförsöksetiska Nämd, ethical permit N226/15 and N5/14) and conducted according to The Swedish Animal Agency´s Provisions and Guidelines for Animal Experimentation recommendations. In order to comply with the 3R strategy of animal welfare, we decided to use data generated for previous studies^2, 3^. No additional animals have been used in this study. All experiments on animals were conducted in compliance with the ARRIVE guidelines.

**Figure 1:**
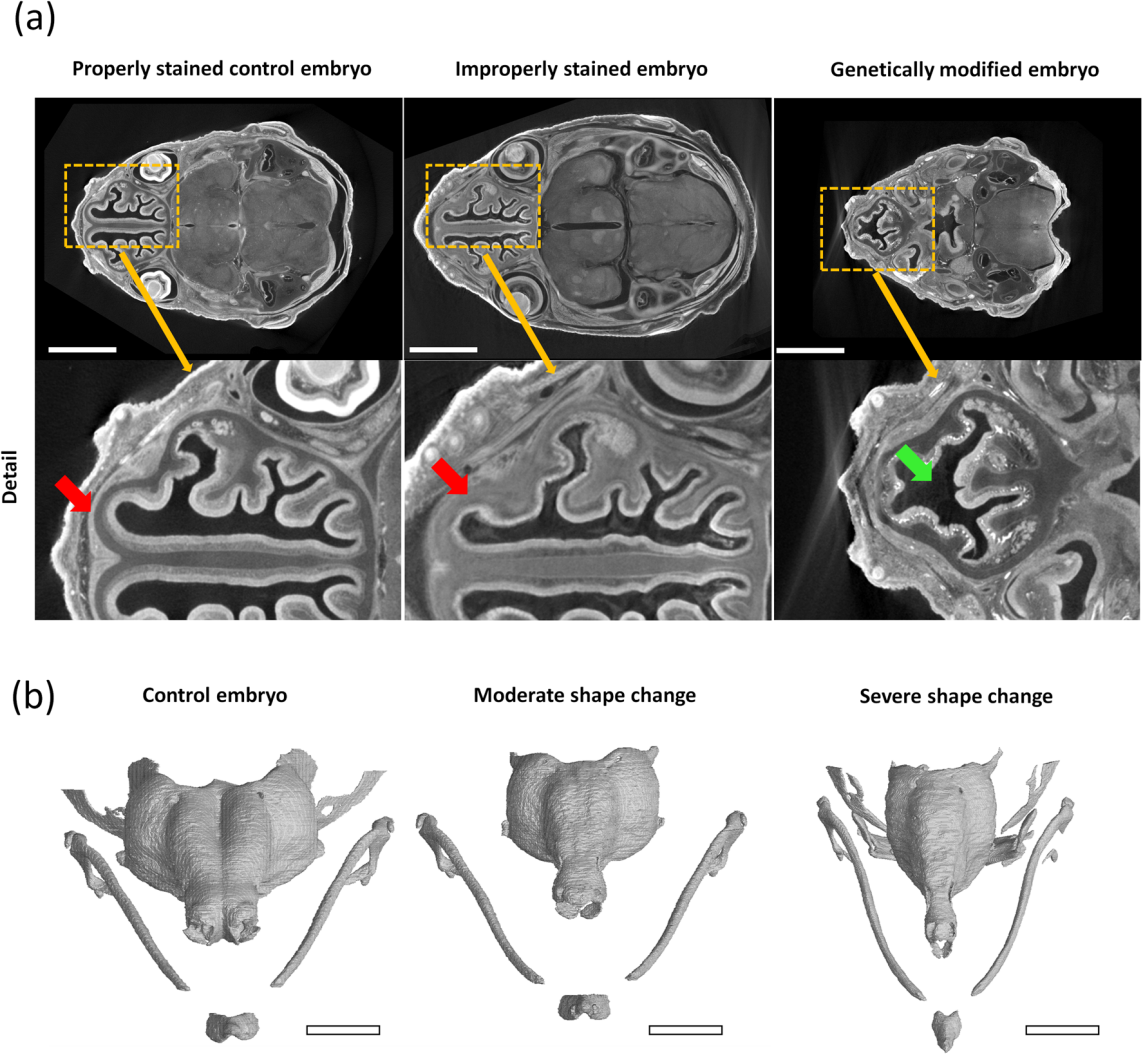
Visualisation of selected samples included in the database utilised in this work. (**a**) Visualisation of challenging cases compared to control mouse embryo represented in the majority of the database. The figure shows selected tomographic cross-sections of properly stained control embryos in comparison with improperly stained embryos. Red arrows indicate the difference in cartilage staining between properly stained samples and an improperly stained sample. A tomographic cross-section of genetically modified sample 10 is also shown. The green arrow shows the main phenotype difference: the underdeveloped nasal septum. Scale bar 2 mm. (**b**) Frontal view of 3-D rendering of the segmented nasal capsule cartilage of the control (Sample 8), moderate shape change in Sample 6 and severe shape change in Sample 10. Scale bar 1 mm.

Multiple genetically modified embryos with altered cartilage development were included in the database to improve the generalisability of the developed method. As proper sample preparation is very important, we included an improperly stained embryo during the sample preparation procedure. The differences are further visualised in the tomographic cross-section in Fig. 1a. The changes in the cartilaginous nasal capsule geometry and morphology in genetically modified samples differ in severity from moderate to severe. The shape differences found in mutant embryos are visualised as 3-D renders in Fig. 1b.

### Sample preparation

Mice were sacrificed with isoflurane (Baxter KDG9623) overdose or cervical dislocation, and embryos were dissected and collected in ice-cold PBS. Subsequently, the samples were fixed in 4% paraformaldehyde (PFA) in PBS solution for 24 h at + 4 °C with slow rotation. Before contrasting, samples were dehydrated in incrementally increasing ethanol concentrations (30%, 50%, 70%), one day in each concentration to minimise the shrinkage of the tissue. Samples were transferred into 1.5% PTA (phospho-tungstic acid) in 90% methanol for tissue contrasting. The PTA-methanol solution was changed every 2–3 days. Samples were stained for seven weeks. The contrasting procedure was followed by rehydration of the samples by incubation in an ethanol series (90%, 70%, 50% and 30%).

### μCT measurement

The samples were scanned with a laboratory μCT system GE Phoenix v|tome|x L 240 (Waygate Technologies GmbH Germany). The system was equipped with a high contrast flat panel detector DXR250 with 2048 × 2048-pixel resolution and 200 × 200 μm^2^ pixel size. The embryos were fixed in polyimide tubes filled with 1% agarose gel to prevent sample movement during the µCT stage rotation. Two thousand projections were acquired with an exposure time of 900 ms per projection. Each projection was captured three times, and an average of the signal was used to improve the signal-to-noise ratio. The acceleration voltage of the X-ray tube was 60 kV, and the tube current was 200 μA. The X-ray beam was filtered with a 0.1 mm aluminum plate. Tomographic reconstruction of the obtained set of projections was performed using the FDK reconstruction algorithm^27^ in GE phoenix datos |× 2.0 3D computed tomography software (Waygate Technologies GmbH Germany). Output of the reconstructed CT slices was 16-bit integer. To compensate for small and smooth drift of axis (samples and detector) and focus (X-ray tube) position, scan optimiser module was applied during the reconstruction. Beam hardening correction was applied by the commercially available module in the reconstruction software with parameter 7 for different materials. The voxel size was variable depending on the sample size (see Suppl. Table S1 for complete information).

### Manual segmentation

**Figure 2:**
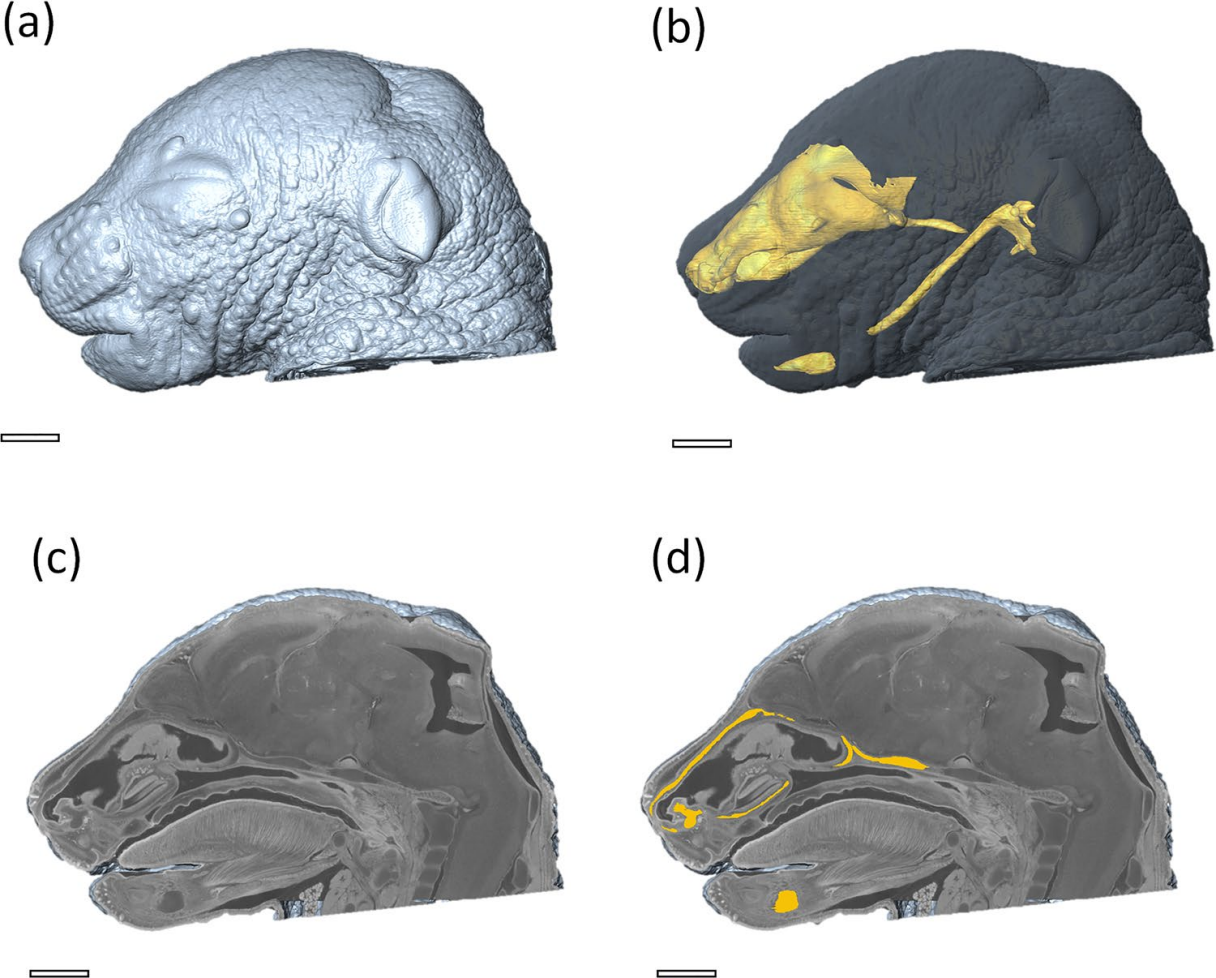
Visualisation of the segmented structure of interest in the context of the mouse embryo head. (**a**) 3-D rendering of the embryo head, (**b**) 3-D rendering of craniofacial cartilage tissue (yellow) in the context of the whole embryo head (grayscale), (**c**) clipping plane through the 3-D rendering showing the tomographic data, and (**d**) yellow showing the manually segmented craniofacial cartilage tissue. Scale bar 1 mm.

Avizo image processing software (version 7, Thermo Fisher Scientific, USA) was used to manually segment the nasal capsule cartilage in the reconstructed CT images. The data were aligned for each embryo head to have the same orientation. The manual segmentation of the cartilaginous nasal capsule tissue takes at least 8 h^16^, depending on the sample and operator’s experience. As a result of the cartilage being segmented by multiple operators, some intraoperator variability is introduced into the manually segmented samples. It was partially avoided by the quality check performed by a single expert, but it might still affect the quality of the dataset and then further evaluation of the segmentation accuracy. To make the load of 3D segmentation volume easier to handle, only every 3^rd^ slice was manually segmented, and the remaining slices were calculated by linearly interpolating between adjacent manually segmented slices. Figure 2 depicts the segmented structure in the context of the whole head in 3-D volume.

### Neural network architecture

We aimed to fully preserve the resolution provided by the µCT imaging modality. In the CNN architecture design, we had to keep in mind the large size of the segmented images, which is over 1000 voxels in all three dimensions. Utilising a fully 3-D CNN architecture for the segmentation of image data of this size is not feasible due to memory limitations. A piecewise segmentation of patches extracted from the 3-D volume seems to be a possible solution to this problem; however, even the segmented structure is enormous for a typical segmentation via 3-D CNN (see Suppl. Table S1). The size of the segmented structure is in each case over 700 × 1000 × 600 pixels. By extracting patches from the whole 3-D volume, much of the global spatial context needed for proper localisation and segmentation of the cartilage would be lost. For these reasons, a slice-by-slice approach to segmentation is the most appropriate. Manual segmentation was performed in the axial slices of the whole 3-D volume, and we thus decided to utilise the axial plane for training and subsequent inference of the developed segmentation model.

**Figure 3:**
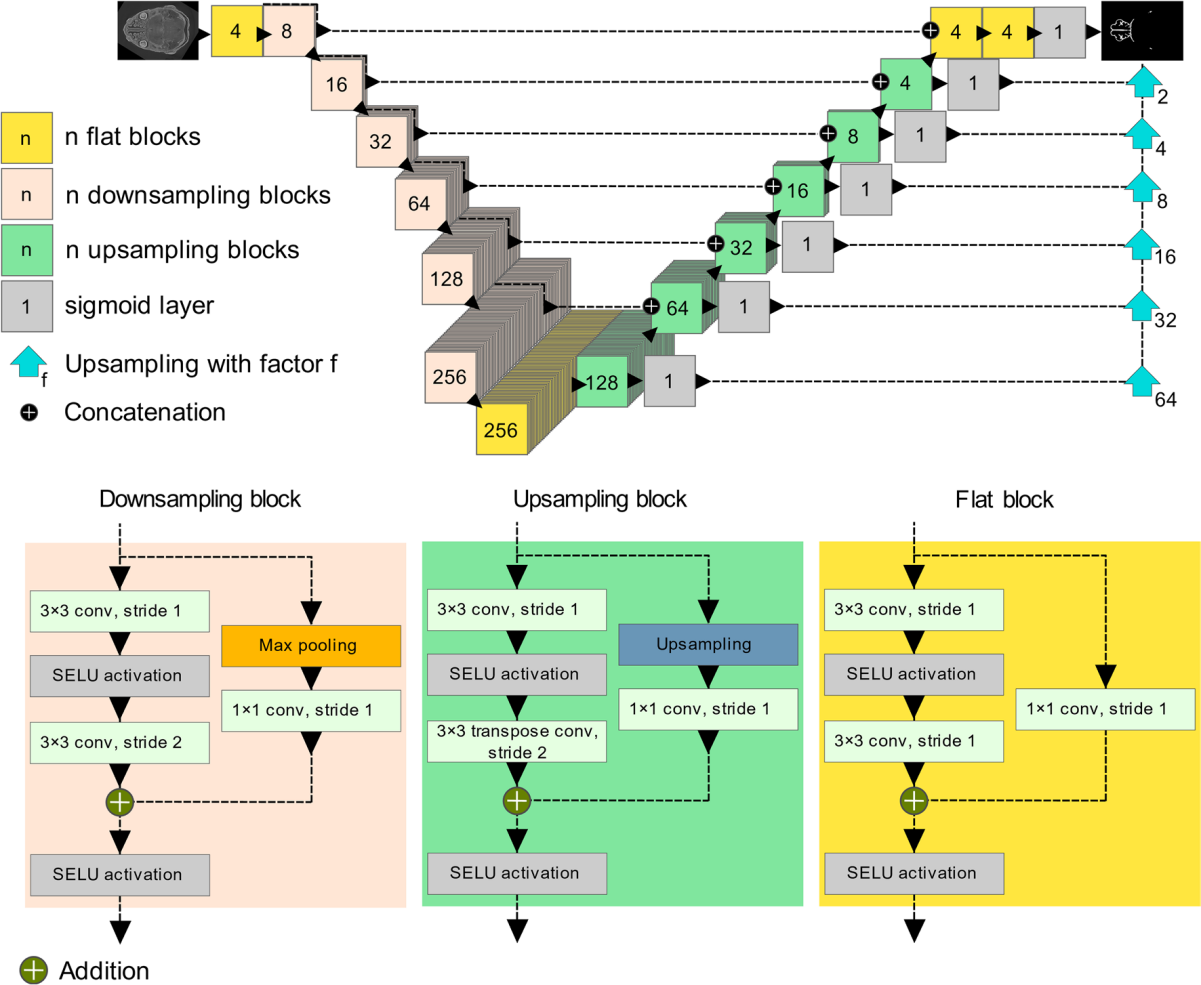
The proposed CNN architecture for segmentation of nasal capsule cartilage.

We use the basic U-Net shape; however, the input is downsampled only four times in the original implementation^12^. To compensate for the large image size, two additional levels were added to the architecture. This means that the input, set to a fixed size of 1792 × 1280 pixels, is downsampled a total of 6 times to the size of 28 × 20 pixels in the lowest level of the network. This makes the network very deep, and issues such as the vanishing gradient could significantly hinder the training of the model. For this reason, the architecture was enhanced by utilising residual blocks that had been first proposed in^20^. Residual blocks are a structure consisting of stacked layers utilised in CNNs. They improve the information flow through the deep network, prevent vanishing gradient problems, and improve the network's training^28^. Three types of residual blocks are used in the architecture (see Fig. 3). A downsampling residual block implements dimensionality reduction in the encoding part of the CNN architecture. Strided convolutional layers achieve dimensionality reduction in the convolutional path of the residual block and max-pooling in the identity path of the residual block. Because in U-Net-based architectures, the number of filters increases twice with each dimension reduction level, it is also necessary to increase the number of filters in the identity part of the residual block. This is performed by a 1 × 1 convolutional layer with the required number of filters to perform the addition of the feature maps from the convolutional and identity paths. Another type of residual block in the proposed architecture is a so-called flat block that outputs feature maps with the same dimensions as the output. The third type of residual block utilised in the proposed architecture is an upsampling block. The upsampling block is a residual equivalent of the transposed convolutional layers of the decoder part of the basic U-Net architecture. The upsampling is performed by transpose convolutional layers in the convolutional path of the residual block and by nearest neighbor interpolation in the identity path. The 1 × 1 convolutional layer in the identity path ensures the correct number of feature maps for the addition with the feature maps from the convolutional path. As in any U-Net-based architecture, feature maps from the encoder are concatenated with the decoder feature maps. The overall CNN architecture is visualised in Fig. 3.

Furthermore, we used the SELU activation function^29^ with LeCun normal weight initialisation in the proposed CNN architecture^30^. SELU is designed by its authors to have a so-called self-normalizing property which makes the training of the network more stable implying better network´s performance. A great advantage of SELU over the other normalization techniques is no need for hyperparameter tuning as well as no dependency on the mini-batch size. To support weight updates even in the deepest part of the network, additional paths were added to each upsampling block: a 1 × 1 convolution layer with a sigmoidal activation function followed by a basic upsampling layer that transforms the feature map dimension to the dimensions of the ground-truth mask. The losses were weighed by the following weights from the deepest layer to the shallowest: 0.03, 0.05, 0.08, 0.12, 0.15, 0.2, and 0.37, with the largest weights being given to the layers with the feature maps of largest dimensions.

## Experiments

### Implementation

The proposed CNN was implemented in the programming language Python (version 3.7.9) using the library Keras^31^ (version 2.3.1) with the TensorFlow backend^32^ (version 2.1.0). CUDA (version 10.1) and CUDnn (version 7.6.5) were used for GPU acceleration of the training and inference process. NumPy^33^, scikit-image^34^ and Pillow libraries were used for manipulating and transforming the image data.

### Data preparation

As the proposed CNN architecture requires a fixed size input, the CT images' dimensions and corresponding manual segmentation masks had to be unified. First, we rescaled the data to a unified voxel size of 6 μm by bilinear interpolation. A suitable dimension size proved to be 1792 × 1280 pixels. This value allowed us to crop the tomographic cross-sections in the case of larger datasets without any loss of relevant information. In the cases where one or both dimensions of the data were smaller than this value, the image data were padded with zero-value pixels. Such prepared data were standardised to 0 mean and standard deviation 1.

### Training

For better generalisation of the trained segmentation model, a custom augmentation procedure is proposed. The augmentation consists of random rotation, vertical flipping, elastic deformation, gamma transform with random parameter gamma, and random scaling (see Table 1) for the transform parameters). Each training image has a certain probability of undergoing two consecutive augmentation transforms. These probabilities are shown in Table 2. The network is trained with the Adam optimisation algorithm^35^ with an initial learning rate of 1e−4, and AMSgrad enabled to improve convergence^36^. Dice loss is utilised^37^. The CNN is trained with a batch size of 4. A nVidia Quadro P5000 with 16 GB of graphical memory was utilised to train the CNN on a system equipped with 512 GB of RAM and an Intel® Xeon® Gold 6248R CPU.

**Table 1:** Augmentation parameters.

	Parameter range
Random rotation	− 10° to 10°
Vertical flipping	–
Random gamma transform	0.9–1.1
Random elastic	–
Random scaling	0.9–1.1

**Table 2:** Augmentation transform probabilities.

Transform 1	Transform 2					
	Random rotation	Vertical flipping	Random gamma	Random elastic	Random scaling	No transform
Random rotation	0.01	0.02	0.01	0.03	0.02	0.01
Vertical flipping	0.02	0.04	0.02	0.06	0.04	0.02
Random gamma	0.01	0.02	0.01	0.03	0.02	0.01
Random elastic	0.03	0.06	0.03	0.09	0.06	0.03
Random scaling	0.02	0.04	0.02	0.06	0.04	0.02
No transform	0.01	0.02	0.01	0.03	0.02	0.01

### Performance evaluation

The performance of the proposed segmentation method was evaluated using the Dice similarity coefficient (DSC). DSC is a generally utilised binary segmentation mask overlap measure. Its maximum value is 1, which signifies a complete overlap of the evaluated segmentation mask and the ground-truth mask^38^. The equation for computing the Dice coefficient from true positive (TP), true negative (TN), false positive (FP) and false negative (FN) segmented pixels can be seen in Eq. 1. A sevenfold cross-validation was performed to evaluate the accuracy of segmentation with the proposed model. This means that the model was trained on 12 samples and evaluated on the remaining two.

1 $$DSC=\frac{2*TP}{2*TP+FP+FN}$$

### Ablation experiment

To show the benefits of the proposed modifications to the base U-Net-shaped CNN architecture, we performed an ablation experiment. For each individual ablation, we eliminated one of the proposed modifications from the CNN segmentation methodology. These modifications are: residual blocks, deep supervision, SELU activation, increased depth and the proposed augmentation techniques. Visualisations of the CNN architectures used for the ablation experiment can be found in the Supplementary material S1 of this work (supplementary Figs. S1–S4). To make the ablation experiment less time demanding, only a subset of the training database is used for the experiments. Every 200th tomographic cross-section not containing cartilage and every 30th cross-section from the region containing cartilage tissue is used from each sample. Other than these modifications to the methods, the remaining hyperparameters are kept identical to the hyperparameters outlined in the Training section of this chapter. We again performed the ablation experiment as a sevenfold cross-validation, where the models were trained on 12 samples and validated on the remaining two. The model from the epoch where the lowest validation loss was achieved was used for the cross-validation.

### Wall thickness analysis

Wall thickness analysis was performed using VG Studio MAX 3.5 software (Volume Graphics GmbH, Germany). The wall thickness for each voxel was calculated as the diameter of the largest inscribed sphere to the volume, which still contains the center position of the voxel.

## Results and discussion

**Figure 4:**
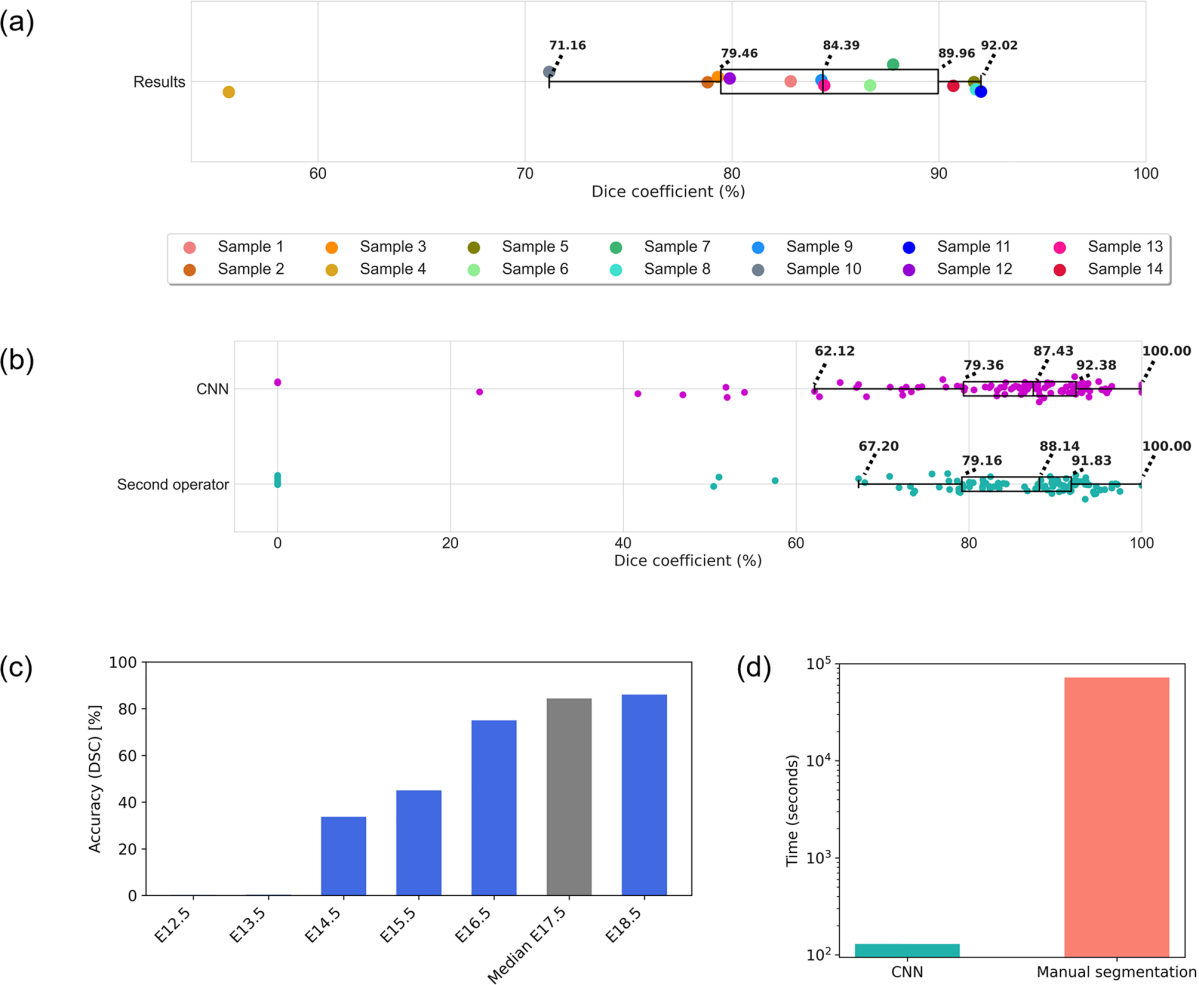
Evaluation of the segmentation accuracy of the proposed image segmentation model. (**a**) Segmentation accuracy boxplot. The box extends from the first quartile *Q*1 to the third quartile *Q*3, and its length represents the interquartile range (*IQR* = *Q*3 − *Q*1). The length of whiskers is the largest and smallest data point lying within the range defined by 1.5·*IQR* subtracted from *Q*1 and added to *Q*3. The line inside the box represents the median. (**b**) Time requirements comparison of the CNN and manual segmentation for segmentation of one mouse embryo scan. (**c**) The accuracy of segmentation with the CNN trained on the available database of 17-day-old embryos applied for segmentation of the nasal capsule in images of mouse embryos in other developmental stages. (**d**) Time requirements comparison of the CNN and manual segmentation for the segmentation of one mouse embryo scan.

The results of the sevenfold cross-validation are summarised in Table 3. The results of the segmentation were compared with the ground-truth segmentation masks via the Dice coefficient. The results are also visualised in the form of a boxplot (Fig. 4a), where each point represents the segmentation accuracy of a 3-D segmented sample.

**Table 3:** Results of the sevenfold cross-validation.

Sample code	Dice coefficient [%]	Comment
Sample 1	82.8282	Control
Sample 2	78.8173	Control
Sample 3	79.3120	Control
Sample 4	55.6860	Improper staining
Sample 5	91.6862	Control
Sample 6	86.6735	Genetically modified
Sample 7	87.7789	Genetically modified
Sample 8	91.7748	Control
Sample 9	84.3186	Control
Sample 10	71.1617	Genetically modified
Sample 11	92.0235	Control
Sample 12	79.8854	Control
Sample 13	84.4525	Control
Sample 14	90.6867	Control

**Figure 5:**
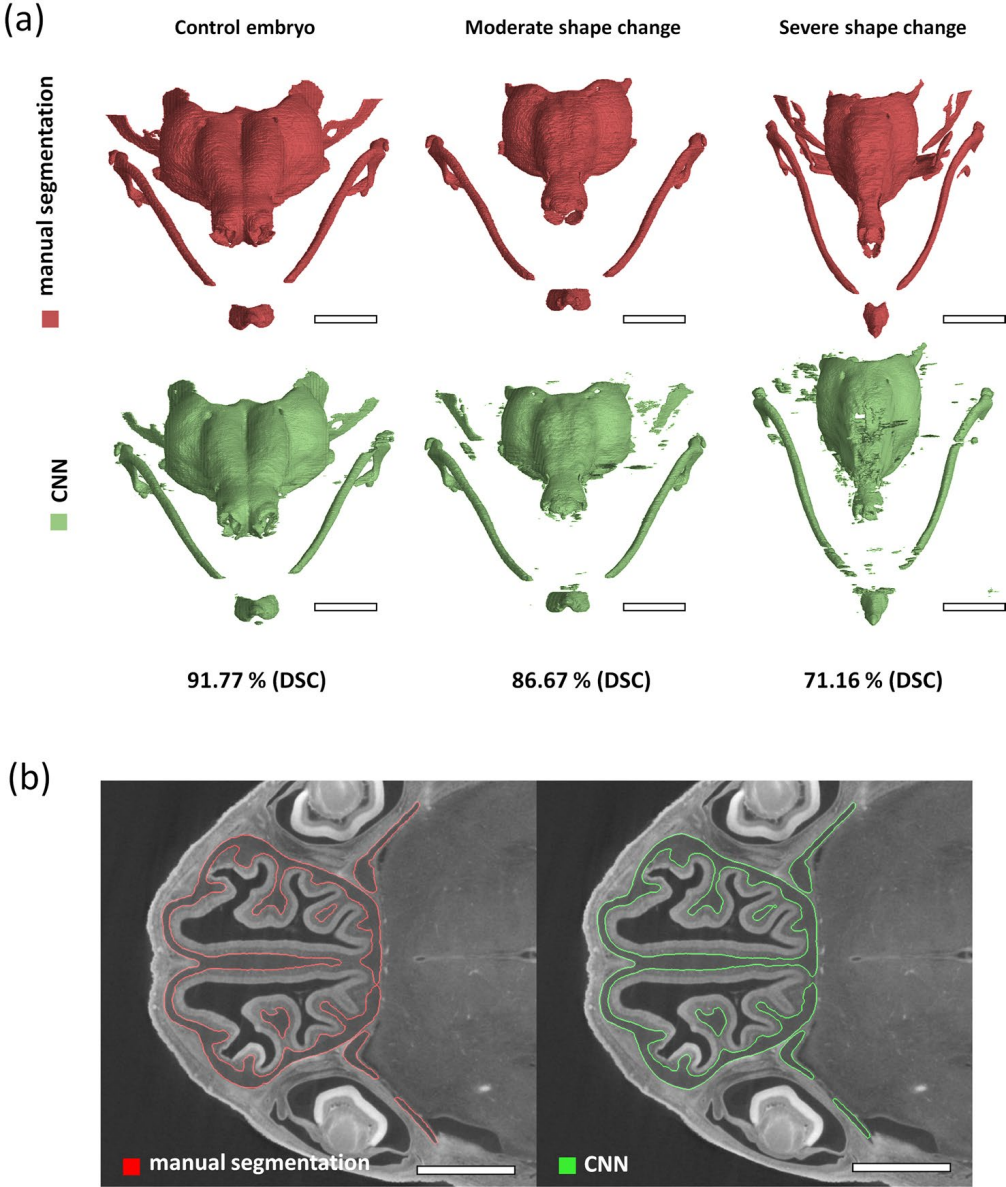
Visualisation of the proposed segmentation model’s output together with the ground-truth data. (**a**) Comparison of the nasal capsule 3-D renders created by manual segmentation (red) and the CNN (green). Note the decrease in the segmentation accuracy in samples with significantly changed morphology due to genetic modfications. Scale bar 1 mm. (**b**) Visualisation of the proposed segmentation (green contour) in a selected tomographic cross-section of Sample 8 compared to manual segmentation (red contour). Scale bar 1 mm.

According to the Dice coefficient, the median segmentation accuracy is 84.44%, with the largest outlier being Sample 4, with a segmentation accuracy of merely 55.68%. As shown in Fig. 1, Sample 4 was improperly stained during the sample preparation procedure, and the proposed segmentation model could not correctly identify the necessary features for the accurate segmentation of the cartilage. It is thus essential that the staining protocol performed prior to the μCT measurement be followed correctly for the segmentation model to perform well. Sample 10 is a severely affected mutant embryo, significantly different from the rest of the available database. It was included in the training and evaluation of CNN to show its capabilities of processing even morphologically different samples. The DSC of 71.16% is relatively low compared to the remaining database, and more scans of mutant mouse embryos should be included in the training database to improve the model segmentation accuracy of this type of sample. The moderately changed mutant embryo (Sample 6) was segmented with an above-average accuracy of 86.67%. See Fig. 5a for a visualisation of the difference in the segmentation accuracy in genetically modified embryos. Figure 5b then shows an example of both manual and automatic segmentation in a selected tomographic cross-section of Sample 8.

We also evaluated the proposed method in comparison with 100 randomly selected tomographic slices from the validation fold of the available database, segmented by a second independent operator to see if the proposed CNN behaves similarly to an independent human operator performing the manual segmentation. The segmentation was performed the same way as the segmentation of the ground-truth data: Avizo (Thermo Fisher Scientific, USA) was used. Both the CNN segmentation and the independent operator segmentation were compared with the ground-truth segmentation masks using the Dice coefficient. The results of this experiment are summarised in Fig. 4b in the form of a boxplot, where each data point represents one segmented tomographic slice. The median accuracy of the automatic CNN segmentation with respect to the ground-truth data was 87.43%, and the accuracy of the second operator with respect to the ground-truth data was 88.14%. There was also a moderate positive correlation between the values (Spearman coefficient 0.59, p < 0.01). This shows that the CNN operates within the scope of the intraoperator variability. As such, the segmentation error might be caused partially by the uncertainty of the manual segmentation in some regions of the cartilage.

The performance of the trained segmentation model was also evaluated on samples from different developmental stages that were not present in the training database (specifically embryos from the 12th to 18th day of their development). The accuracy of such segmentation was 86% (DSC) for the sample on the 18th day of development (E18.5) and 72% for the scan of embryos on the 16th day of development (E16.5). We performed Theiler staging of the embryos in this external dataset.^25, 39^ Theiler stages objectively evaluate the development of the embryos based on their morphology independently on their gestational age. The Theiler stages for both the 17 day old embryo and 18 day old embryo is the same (Theiler stage 26), with 16 day old embryo being only 1 stage lower (Theiler stage 25). These samples were not involved in the development of the proposed method and these results thus show, that the proposed methodology performs well even on an external test set of embryos with comparable developmental stages. When the network is applied to earlier stages, the segmentation accuracy decreases rapidly. In the developmental stages from 12 to 13 days, when the cartilage is not fully developed and mesenchymal condensations are still present, the trained CNN fails completely (see Fig. 4c). Including other developmental stages in the training database might improve the robustness of the method; however, using the same segmentation model to segment the images of embryos in earlier developmental stages than 14 days after conception, before the cartilage is formed, seems not feasible.

**Figure 6:**
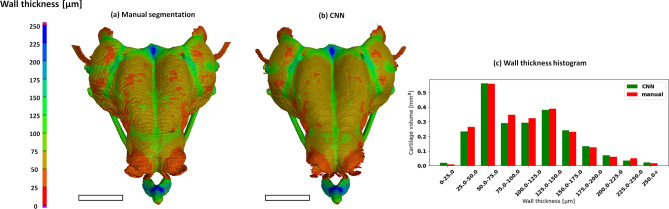
Visualisation of wall thickness analysis applied to the 3-D model created by manual segmentation (**a**) and by the proposed automatic segmentation (**b**). Scale bar 1 mm. (**c**) The histogram of the wall thickness distribution.

As a further qualitative check of the segmentation accuracy, we performed a wall thickness analysis of the segmented structure for both the 3-D model created by manual segmentation and the 3-D model created by the proposed CNN (Fig. 6). Wall thickness analysis is a routine follow-up analysis to show additional developmental changes. Figure 6 shows the wall thickness analysis of Sample 8. As the wall thickness histogram (c) in Fig. 6 shows, the results of wall thickness analysis performed on both 3-D models are very similar. This is also demonstrated by the very high positive correlation of the wall thickness distributions (Spearman coefficient 0.98, p < 0.01). Slight differences may be caused by the step artefact produced by the manual segmentation performed only in a single plane. Even though the CNN also performs segmentation in a single plane, its predictions are much smoother.

**Figure 7:**
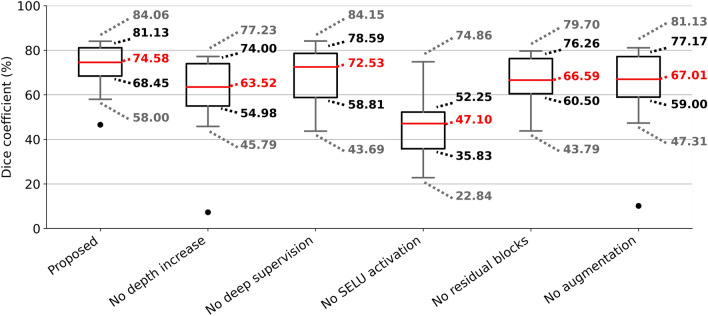
Results of the ablation experiment. Each box represents sevenfold cross-validation segmentation accuracy of a CNN trained without one of the modification to the architecture or training strategy employed in this work. The boxes extend from the first quartile *Q*1 to the third quartile *Q*3, and its length represents the interquartile range (*IQR* = *Q*3–*Q*1). The length of whiskers is data largest and smallest data point lying within the range defined by 1.5·*IQR* subtracted from *Q*1 and added to *Q*3. Outliers are marked as a black dot.

We performed an ablation experiment to evaluate the contribution of each proposed modification to the CNN architecture and to the training strategy towards the total nasal capsule cartilage segmentation accuracy. Here we removed the modifications from the complete architecture and one by one evaluated the segmentation accuracy of each model by sevenfold cross-validation. The results of this experiment can be seen in Fig. 7. The proposed methodology employing increased depth of the CNN, deep supervision, SELU activations, residual blocks and the proposed image augmentation strategy provides the highest median segmentation accuracy: 74.58% (DSC). Note that this number is significantly lower than the median segmentation accuracy presented in Fig. 4a. This lower segmentation accuracy is caused by training the CNNs in the ablation experiment on a reduced training set of images to make the ablation experiment less time-demanding. Deep supervision seems to provide only minor improvement to the total segmentation accuracy, as the median segmentation accuracy is lower only by ~ 2% (DSC) when training without deep supervision. Training models without utilising the residual blocks or the proposed augmentation procedure sees a more significant drop in the cross-validation accuracy to the median of ~ 67% (DSC). This justifies the use of residual blocks to improve the training of the CNN. The segmentation accuracy of the CNN without the increased depth drops even further to the median of 63.52% (DSC). This decrease in segmentation accuracy is expected as the shallow network has fewer trainable parameters and cannot benefit from the abstract features extracted in the deep layers of the proposed CNN. Finally, the most significant drop in accuracy is observed when not substituting the ReLU activations for SELU activations. This shows that the reported self-normalizing property of the SELU activation function dramatically improves the final segmentation accuracy and generalisability of the trained image segmentation model. This makes SELU an extremely valuable addition to the CNN architecture.

As in many supervised machine learning application tasks, the performance and generalisability of the trained model are closely tied to the distribution of the training database. In our work, the proposed CNN was trained exclusively on data originating from a single μCT scanner with the samples measured under a unified methodology (sample staining, scanning parameters, resolution, image size). The methodology described here should be followed as closely as possible to achieve segmentation performance comparable to the results shown in this work. We artificially enlarged the training database by applying selected data augmentation techniques; however, despite this fact, a decrease in performance should be expected when deviating from the outlined data acquisition methodology. This decrease in segmentation accuracy was demonstrated in the case of Sample 4, where the staining of the sample is significantly different from the rest of the database. Expanding the training database by adding a more significant number of samples coming from different CT systems and obtained under different conditions concerning sample preparation and measurement parameters could dramatically improve the generalizability of the segmentation model. Such a database is unfortunately not yet available for this particular segmentation problem. It would, however, be highly beneficial to utilise the weights of the trained CNN as a starting point for training a nasal capsule cartilage segmentation model on new data obtained with significantly different parameters, as the basic extracted features used to predict the cartilaginous nasal capsule will always be similar. This type of transfer learning could significantly improve the convergence of the segmentation model to an optimum with a lower training time.

## Conclusion

In this work, we have demonstrated a highly efficient and time-saving application of a custom U-Net-based CNN for the segmentation of cartilaginous tissue in μCT images of mouse embryos. We employed this architecture and trained it on a database of 14 3-D manually segmented μCT scans. It has been proven that a highly accurate, fully automatic segmentation (84.44% overlap with ground truth according to the Dice coefficient) of the complex cartilaginous structures in a developing mouse head is achievable via deep learning and will be vital for accelerating research on mammalian chondrocranium. One of the primary motivations for this work was to reduce the time required to process new data by employing a fully automatic segmentation procedure instead of the time-demanding manual segmentation. Training of the model on 12 samples for 50 epochs took approximately 27 h. The model is then able to segment a new sample in approximately 130 s (Fig. 4d), depending on the number of tomographic cross-sections present and on available hardware. This segmentation model will be further used to segment new samples, including models of major congenital craniofacial and skeletal diseases. It is possible to obtain an even larger training database by manual corrections of the initial segmentation results and make the final model even more robust.

## Supplementary Information

Supplementary Information.

## Data Availability

Due to the training data coming from multiple sources and studies, it is currently not feasible to share the complete training and testing database; however, a subset of the whole database was published as an X-ray microtomography-based atlas of mouse embryo cranial development and can be accessed at^26^. The trained models and accompanying code can be found in a public GitHub repository: https://github.com/janmatula/deep-mouse-cartilage.

## Abbreviations

μCT: X-ray computed microtomography
CNN: Convolutional neural network
ReLU: Rectified linear unit
SELU: Scaled exponential linear unit
DSC: Dice–Sørensen coefficient
TP: True positive
TN: True negative
FP: False positive
FN: False negative
IQR: Interquartile range
