# Supplementary material for: Resolving complex cartilage structures in developmental biology via deep learning-based automatic segmentation of X-ray computed microtomography images
Source: Sci Rep. 2022 May 24;12:8728. doi: 10.1038/s41598-022-12329-8 (PMC9130254; doi:10.1038/s41598-022-12329-8)
Supplement: Supplementary file 1 — Supplementary Information. [file 41598_2022_12329_MOESM1_ESM.pdf]

Supplementary information: Resolving complex cartilage structures in developmental biology via deep learning-based automatic segmentation of X-ray computed microtomography images

Jan Matula[1], Veronika Polakova[1], Jakub Salplachta[1], Marketa Tesarova[1], Tomas Zikmund[1], Marketa Kaucka[2], Igor Adameyko[3], *Jozef Kaiser[1]

[1] Central European Institute of Technology, Brno University of Technology, Purkynova 123, Brno, 61200, Czech Republic

[2] Max Planck Institute for Evolutionary Biology, August-Thienemann-Str.2, 24306 Ploen, Germany

[3] Medical University of Vienna, Spitalgasse 23, Vienna, 1090, Austria

* corresponding author

Suppl. Table 1: List of samples

Sample code	Image dimensions	Mask bounding box dimensions	Voxel size	Phenotype
Sample 1	1158 × 1637 × 1257	762 × 1047 × 651	6.2	wild type
Sample 2	1478 × 2073 × 1379	908 × 1145 × 809	5.7	wild type
Sample 3	1372 × 1936 × 1436	873 × 1167 × 750	6	wild type
Sample 4	1267 × 1946 × 1386	857 × 1165 × 722	6	wild type
Sample 5	1262 × 1906 × 1462	855 × 1122 × 739	5.6	wild type
Sample 6	1340 × 1854 × 1395	939 × 1063 × 764	5	mutant
Sample 7	1346 × 1786 × 1409	922 × 1049 × 702	5	mutant
Sample 8	1244 × 1783 × 1158	842 × 1104 × 740	5.3	wild type
Sample 9	1344 × 1801 × 1344	806 × 1059 × 678	5.8	wild type
Sample 10	1408 × 1886 × 1536	929 × 1097 × 838	4.5	mutant
Sample 11	1406 × 1961 × 1429	901 × 1129 × 757	5.2	wild type
Sample 12	1990 × 2314 × 1530	856 × 1151 × 437	4.9	wild type
Sample 13	1351 × 1926 × 1441	896 × 1122 × 789	5.7	wild type
Sample 14	1350 × 1934 × 1589	932 × 1182 × 797	5	wild type

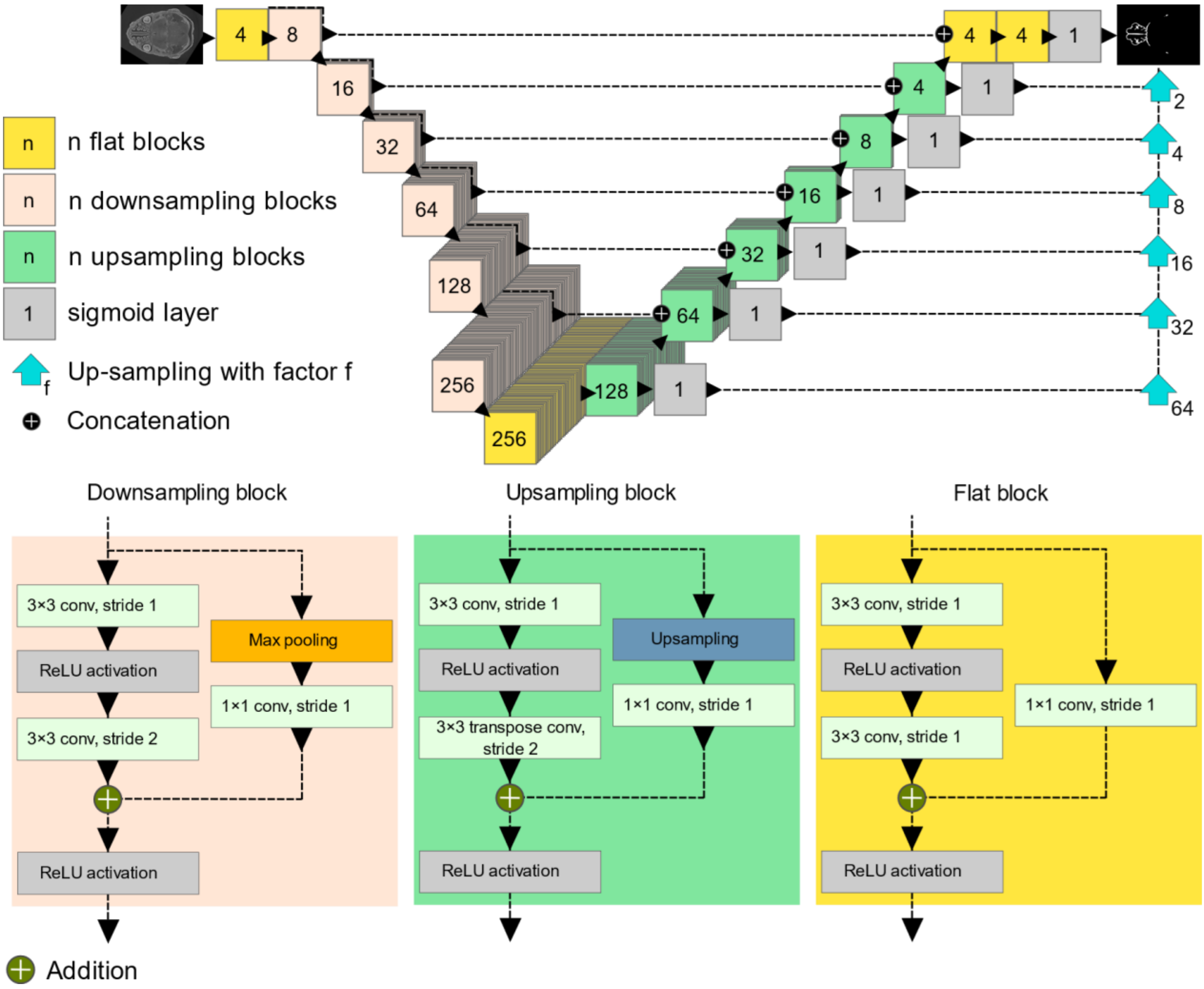

Suppl. Figure 1: CNN architecture used for the ablation experiment without employing SELU activation function.

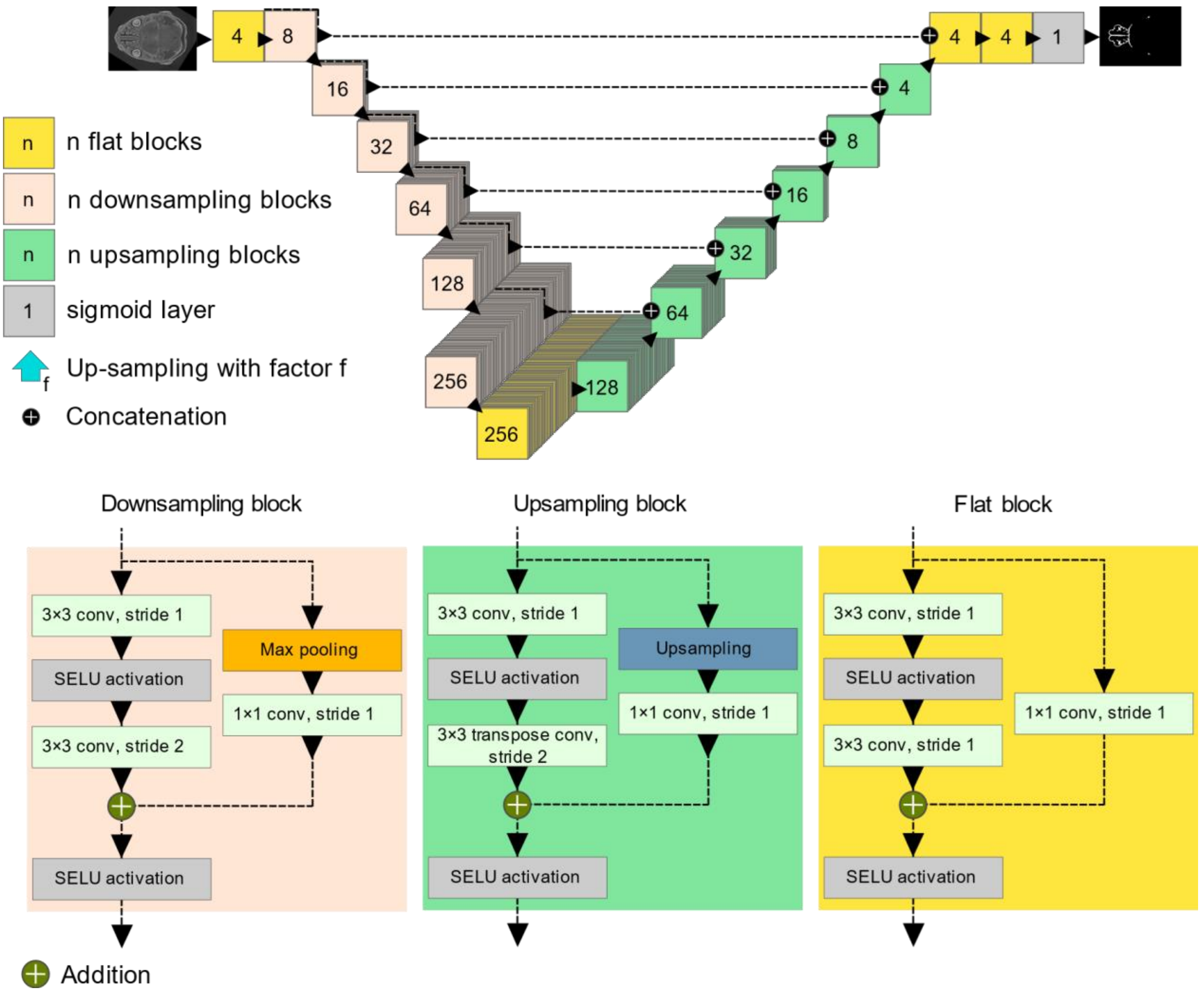

Suppl. Figure 2: CNN architecture used for the ablation experiment without deep supervision.

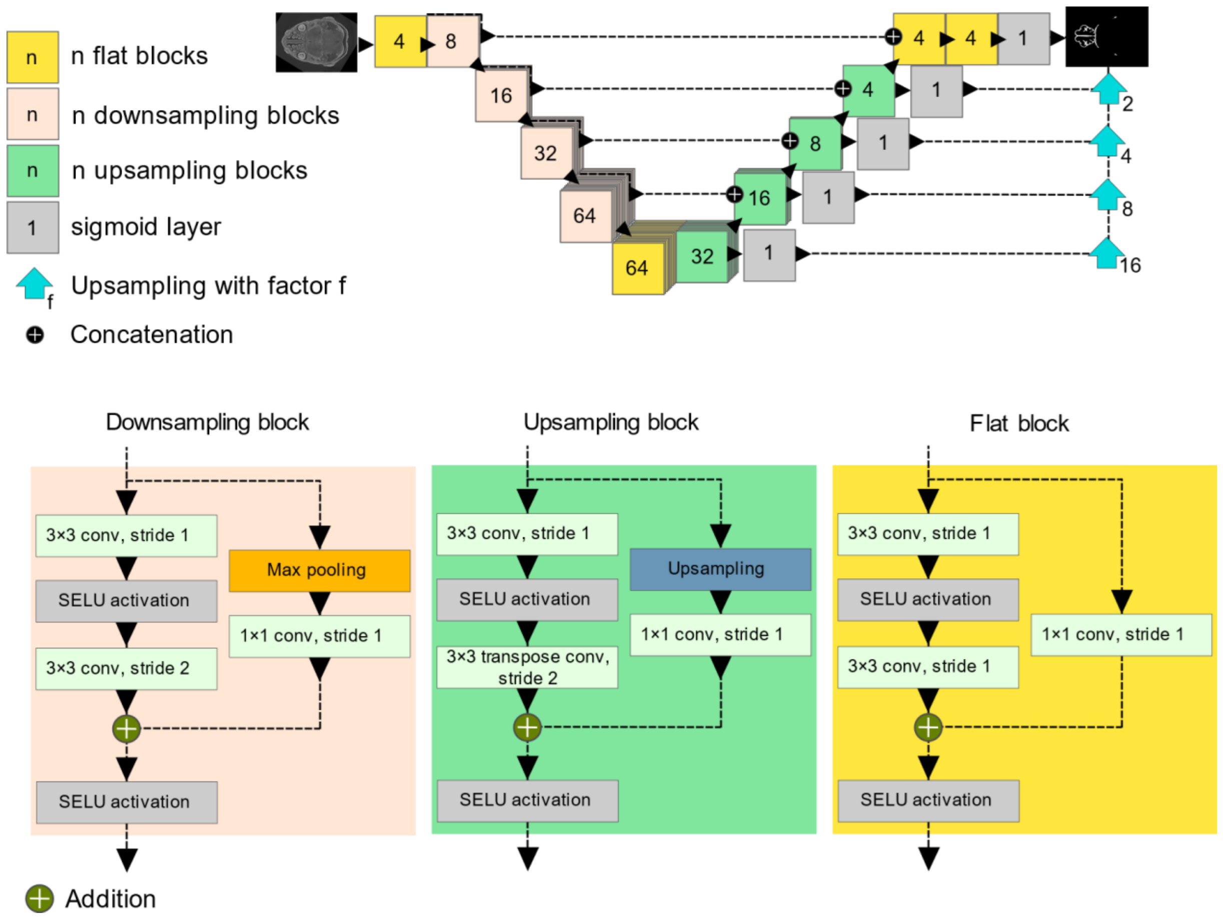

Suppl. Figure 3: CNN architecture used for the ablation experiment without adding two additional levels to the U-net CNN shape.

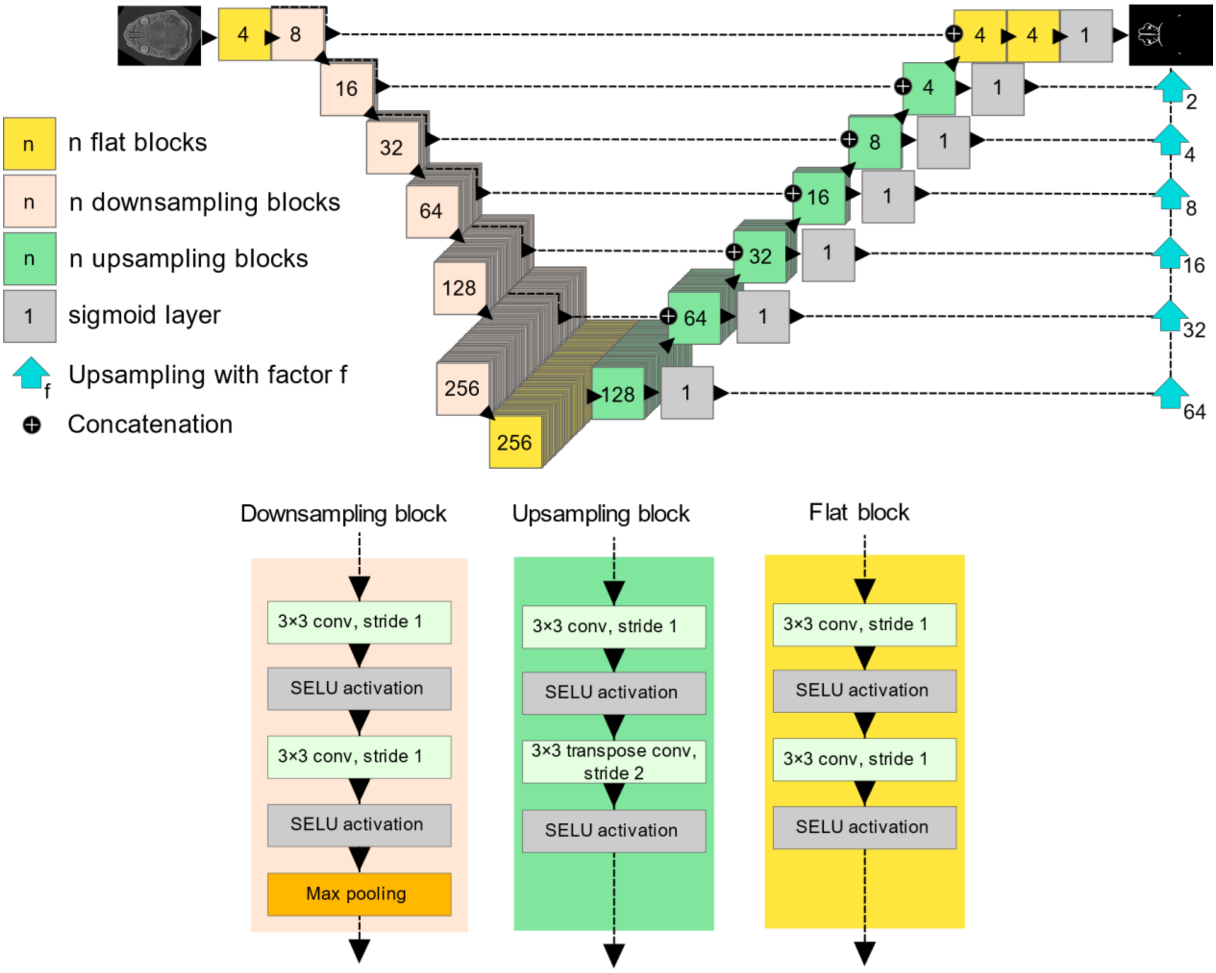

Suppl. Figure 4: CNN architecture used for the ablation experiment without residual blocks.
